# Using Near-infrared reflectance spectroscopy (NIRS) to predict glucobrassicin concentrations in cabbage and brussels sprout leaf tissue

**DOI:** 10.1186/s13007-020-00681-7

**Published:** 2020-10-12

**Authors:** Ilse E. Renner, Vincent A Fritz

**Affiliations:** 1 Department of Horticultural Science, University of Minnesota, Saint Paul, MN 55108 USA; 2 Southern Research and Outreach Center, University of Minnesota, Waseca, MN 56093 USA

**Keywords:** Glucobrassicin, *Brassica oleracea*, Cabbage, Brussels sprouts, Near-infrared spectroscopy, Chemometrics, Partial least squares regression, Chemoprevention

## Abstract

**Background:**

Glucobrassicin (GBS) and its hydrolysis product indole-3-carbinol are important nutritional constituents implicated in cancer chemoprevention. Dietary consumption of vegetables sources of GBS, such as cabbage and Brussels sprouts, is linked to tumor suppression, carcinogen excretion, and cancer-risk reduction. High-performance liquid-chromatography (HPLC) is the current standard GBS identification method, and quantification is based on UV-light absorption in comparison to known standards or via mass spectrometry. These analytical techniques require expensive equipment, trained laboratory personnel, hazardous chemicals, and they are labor intensive. A rapid, nondestructive, inexpensive quantification method is needed to accelerate the adoption of GBS-enhancing production systems. Such an analytical method would allow producers to quantify the quality of their products and give plant breeders a high-throughput phenotyping tool to increase the scale of their breeding programs for high GBS-accumulating varieties. Near-infrared reflectance spectroscopy (NIRS) paired with partial least squares regression (PLSR) could be a useful tool to develop such a method.

**Results:**

Here we demonstrate that GBS concentrations of freeze-dried tissue from a wide variety of cabbage and Brussels sprouts can be predicted using partial least squares regression from NIRS data generated from wavelengths between 950 and 1650 nm. Cross-validation models had R^2^ = 0.75 with RPD = 2.3 for predicting µmol GBS·100 g^−1^ fresh weight and R^2^ = 0.80 with RPD = 2.4 for predicting µmol GBS·g^−1^ dry weight. Inspections of equation loadings suggest the molecular associations used in modeling may be due to first overtones from O–H stretching and/or N–H stretching of amines.

**Conclusions:**

A calibration model suitable for screening GBS concentration of freeze-dried leaf tissue using NIRS-generated data paired with PLSR can be created for cabbage and Brussels sprouts. Optimal NIRS wavelength ranges for calibration remain an open question.

## Background

The genus *Brassica*, which includes commonly consumed vegetables such as cabbage and Brussels sprouts, produce a class of cancer-preventing compounds known as glucosinolates (GSLs). GSL quantification is necessary to determine the health-promoting benefit of fresh vegetables. Current quantification methods are time-consuming, destructive, hazardous, and expensive. Consequently, this limits the timely application of GSL-enhancing technologies. Alternative methods that address these limitations will help usher in new markets for GSL-enhanced vegetables and increase the screening capacity of high-GSL vegetable breeding programs.

GSLs are nitrogen- and sulfur-containing secondary compounds produced by plants of the order Brassicales, and they are particularly abundant in the economically important Brassicaceae family [1]. GSL hydrolysis products are toxic to many pests and thereby protect the plant against herbivores and pathogens [2, 3]. Across all known GSL-containing plants, 88 individual GSLs have been directly identified using nuclear magnetic resonance spectroscopy and mass spectrometry, and the identities of an additional 49 GSLs have been inferred using indirect chemical elucidation methods based on GSL breakdown products [4]. Of these, a handful of individual GSL hydrolysis products have been implicated in cancer prevention [5].

Hydrolysis of GSLs occurs via the endogenous plant enzyme myrosinase (thioglucoside glucohydrolase, EC 3.2.1.147) and results in a suite of bioactive compounds such as isothiocyanates, indoles, nitriles, thiocyanates, epithionitriles, and oxazolidines [6]. The fate of the hydrolyzed compound depends on the parent GSL molecule and the environment during hydrolysis. The indole-3-carbinol compound derived from the parent GSL, glucobrassicin (GBS), has been the subject of chemoprevention research [7]. Dr. Lee Wattenberg, at the University of Minnesota, laid the groundwork for chemoprevention research using vegetable constituents from *Brassica* species. Wattenberg found that mice fed diets rich in Brussels sprouts and cabbage had greater carcinogen detoxification capabilities than mice that ate a non-cruciferous diet [8]. This capability was later attributed to indole-3-carbinol [9]. Indol-3-carbinol has suppressed tumors in mice [10]. It also increased the rate of carcinogen metabolism and excretion in mouse models [11], and this effect was subsequently demonstrated in humans [12]. It is widely thought that increasing GBS consumption could be an effective way to prevent cancer development and progression [13].

GSL profiles and concentrations are affected by genotype, the environment, and genotype by environment interactions [14]. In Brussels sprouts and cabbage, GBS dominates the GSL profile [15, 16], which makes these specific vegetables excellent targets for GBS-enhancing production systems and breeding efforts. Currently, high-performance liquid-chromatography (HPLC) is the standard GSL quantification method [17]. HPLC allows for chemical separation, and chemical quantification is calculated based on the absorbance of a chemical peak multiplied by a response factor of a known concentration of an internal or external standard, or via mass spectrometry. It is expensive, time consuming, requires hazardous chemicals, and is plant destructive. An alternative method, such as near-infrared reflectance spectroscopy (NIRS) analysis, is relatively inexpensive, rapid, does not require hazardous chemicals, and is nondestructive. It does not allow for chemical quantification, and so calibration through chemometric techniques is needed to estimate chemical concentrations.

NIRS is a form of vibrational spectroscopy utilizing wavelengths between 780 and 2500 nm. This radiation causes molecular vibrations especially in C-H, O–H, and N–H bonds. The transmission or reflectance is detected and recorded, and it corresponds to overtone and combination bands of fundamental molecular vibrations [18]. The vibrations are characteristic of specific molecules, however; the bands are usually broad, so it can be difficult to assign specific chemical information to a spectrum. In order to overcome this, special statistical approaches known as chemometrics can be applied to NIR spectra to extract useful information for estimating the reference values. NIRS has developed into a useful agronomic tool largely due to the work of Karl Norris of the USDA. In the 1960′s, Norris designed a pioneering spectrometer to measure moisture, oil, and protein content of agronomic crops [19]. The development of such analytical techniques opened the door for the field of chemometrics which led to the creation of a wide range of NIRS calibration models for high-throughput phenotyping.

Partial least squares regression (PLSR) has been widely applied to NIRS data and is one of the most common chemometric techniques [20]. PLSR has been used successfully to develop calibration models for GSLs in seeds of Brassicaceae using NIRS data [21]. Unlike multiple linear regression which would over-fit a model when the number of factors far outnumbers the number of observations, PLSR can be used in these cases which often arise from spectral data. It also improves upon multiple linear regression due to its ability to handle collinearity between predictor variables and does not assume that the predictors are fixed. PLSR attempts to extract latent factors to account for the maximum variation in the independent variables. From the latent structure, a set of components describing the maximum correlation between predictors and response variables is selected [22] from which to create a linear model. Other methods have also been used for calibration model development of GSLs including modified PLSR which attempts to remove explanatory variables that are irrelevant to the response. The modified PLSR is cited as having lower errors than PLSR [23] but is a less commonly used approach.

NIRS has been applied to measuring GSL concentrations from seed tissue with excellent success, and this is now a common method for seed GSL quantification [24]. Since humans eat leaf tissue of cabbage and Brussels sprouts rather than the seeds, having quantification methods for leaf tissue is more relevant. NIRS quantification of GSLs from leaf tissue however has not been well studied. We are aware of only six publications that reported using NIRS to develop quantitative calibration models for various GSLs in leaf tissue [25, 30]. Only four of these studies reported calibration models for GBS specifically (Table 1). Of those, Sahamishirazi et al. [27] reported a model that had poor performance in broccoli florets. In *B. rapa* leaf tissue, Font et al. [28] were able to determine low or high GBS concentrations for screening purposes. Hernandez-Hierro et al. [29] developed a calibration model for GBS in broccoli that they suggested would be useful for screening, but they did not elaborate further. The most useful calibration model for determining GBS quantitatively was developed by Chen et al. [30] in *B. albograbra* (Chinese kale) leaf tissue at various stages of maturity. The model performance of these published studies is summarized in Table 2. No useful equations have been developed using cabbage or Brussels sprouts leaf tissue, which would be of interest as these varieties tend to produce the highest GBS concentrations, making them targets for breeders focusing on developing high GBS yielding cultivars or for quality assessment purposes in value-added markets. The purpose of this study was to develop a GBS calibration equation from a wide variety of cabbages and Brussels sprouts using the open source software R Statistic.

**Table 1:** Reference GBS concentrations (µmol g^−1^ dry weight) in vegetative tissue in previously reported GBS calibrations

Citation	Tissue	n	Minimum	Maximum	Standard deviation
[27]	Broccoli floret	100	0.21	0.73	0.24
[28]	Rapeseed leaf	115	0.06	3.45	0.83
[29]	Broccoli floret	46	0.68	7.18	1.09
[30]	Chinese kale leaf	145	0*	26*	4.68

*Actual values not reported. Values here are estimated from figures presented in the cited publication

**Table 2:** Model statistics from previously reported GBS calibration models using vegetative tissue with modified PLS regression

Citation	Tissue	Calibration		Cross-validation		
		R^2^_cal_^a^	SEC^b^	R^2^_CV_^c^	SD·SECV^−1d^	Terms^e^
[27]	Broccoli floret	*NR*	0.21	0.11	0.80	6
[28]	Rapeseed leaf	0.50	0.59	0.41	1.29	3
[29]	Broccoli floret	0.89	0.35	*NR*	2.10	7
[30]	Chinese kale leaf	*NR*	*NR*	0.93	3.84	*NR*

^a^Coefficient of determination of the calibration

^b^Standard error of calibration

^c^Coefficient of determination of the cross validation

^d^Ratio of the standard deviation of the reference data to the standard error of the cross validation

^e^Number of terms used in the model selected for cross-validation

*NR* not reported

## Results and discussion

### Reference glucobrassicin (GBS) values

**Fig. 1:**
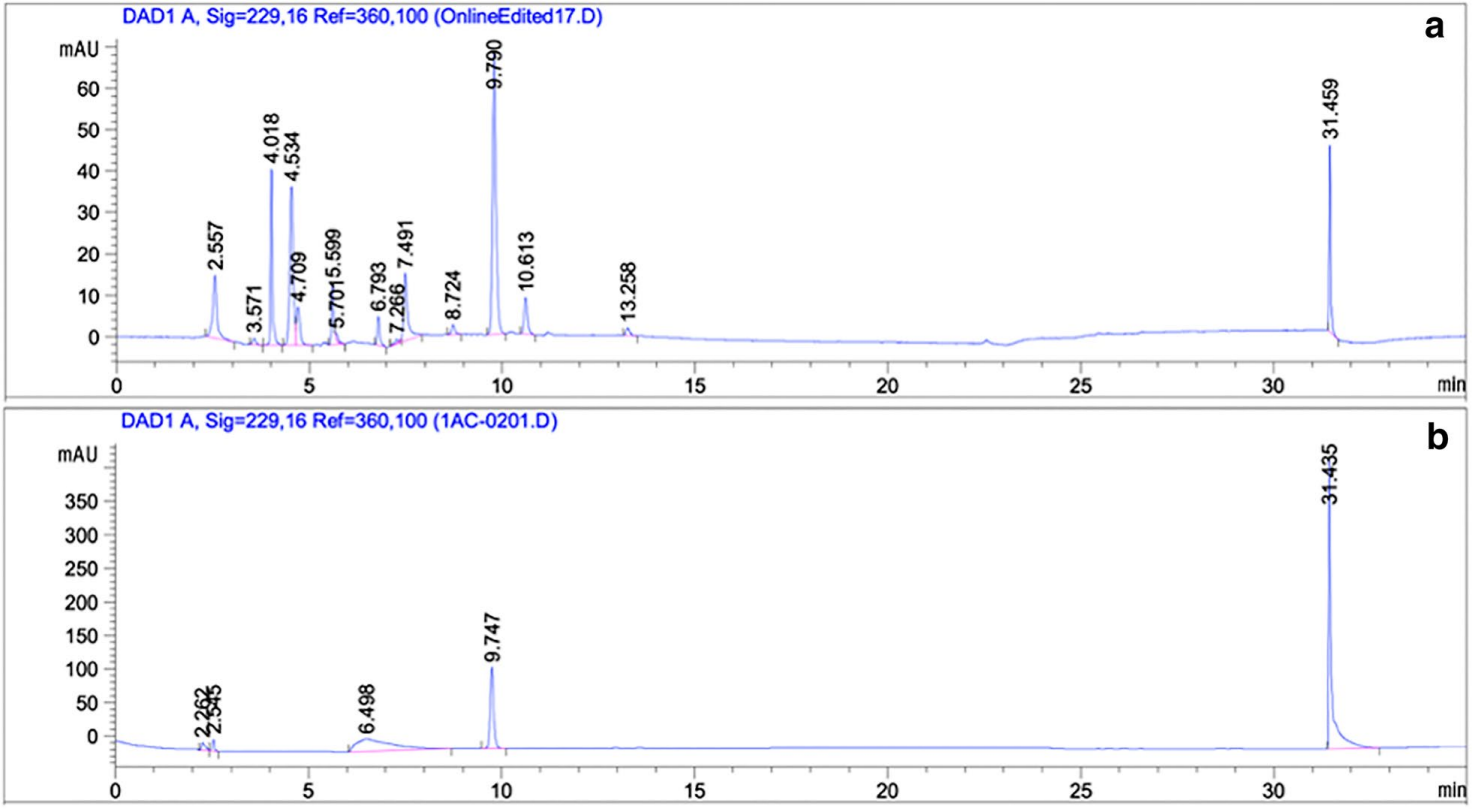
High-performance liquid chromatography chromatogram showing consistent retention time between **a** the presumed glucobrassicin (GBS) peak (9.7 min) from a cabbage sample used in this study and **b** the retention time of a known GBS standard (Product # 80593, PhytoLab, Vestenbergsgreuth, Bavaria, Germany) which was desulphated prior to analysis. Peaks between three and 15 min correspond to individual GSLs. This study was concerned only with GBS, and so the peak at 9.7 min was the only one that was quantified and compared to a standard

**Fig. 2:**
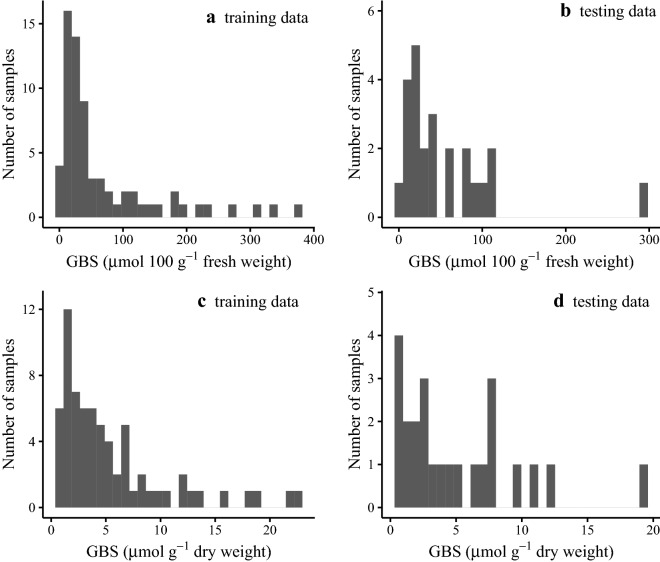
Frequency distributions of the glucobrassicin (GBS) concentrations on a fresh weight basis (µmol·100 g^−1)^ and a dry weight basis (µmol·g^−1^) for the training (n = 68) and testing (n = 24) data subsets used for calibration and cross validation respectively. Samples were derived from cabbage and Brussels sprouts leaf tissue purchased from grocery stores and natural food cooperatives in Minneapolis and Saint Paul, MN. Vegetables were purchased between August and October, 2019. Concentrations were determined using high-performance liquid chromatography and UV-spectroscopy. Plots show the number of samples within a range of GBS concentrations on a fresh weight basis from the **a** training data set and **b** the testing data set and frequency distributions of GBS concentrations on a dry weight basis from the **c** training data set and **d** the testing data set

Models based on both fresh weight and dry weight GBS concentrations were developed. Reference values were attained using desulphated GBS which was identified based on retention time (Fig. 1) and compared to a desulphated GBS potassium salt standard (Product #80593, PhytoLab, Vestenbergsgreuth, Bavaria, Germany). The predominant GSL peak across the samples used for this study was that of GBS. GBS values on a dry weight basis were back calculated based on the fresh weight to dry weight ratio of the sample. The average GBS concentration on a fresh weight basis from a sample size of 92 was 65.16 µmol·100 g^−1^ with a range of 3.69–379.16 µmol·100 g^−1^. The average GBS concentration on a dry weight basis was 5.33 µmol·100 g^−1^ with a range of 0.41 to 22.25 µmol·100 g^−1^. Standard deviations of the reference values were 80.01 µmol·100 g^−1^ and 4.95 µmol·g^−1^ on a fresh weight and dry weight basis respectively. Fresh weight GBS concentrations of the reference values were most abundant at lower concentrations (Fig. 2). GBS concentrations on a dry weight basis had a somewhat improved frequency distribution. This may explain the better performance of the GBS prediction model based on dry weight GBS concentrations. In comparison to the four other studies that developed calibration models for GBS in the typically consumed components of *Brassica* vegetables, we used a wider range of GBS concentrations (Table 3) than the other studies (Table 1) with the exception of Chen et al. [30] who had a similar range but a slightly more right-skewed distribution.

**Table 3:** Reference values of GBS concentration from samples used in this study (n = 92)

Basis of GBS concentration	Minimum	Maximum	Mean	Standard deviation
Fresh weight (μmol GBS·100 g^−1^)	3.69	379.16	65.16	80.01
Dry weight (μmol GBS·g^−1^)	0.41	22.25	5.33	4.95

### Model development

**Fig. 3:**
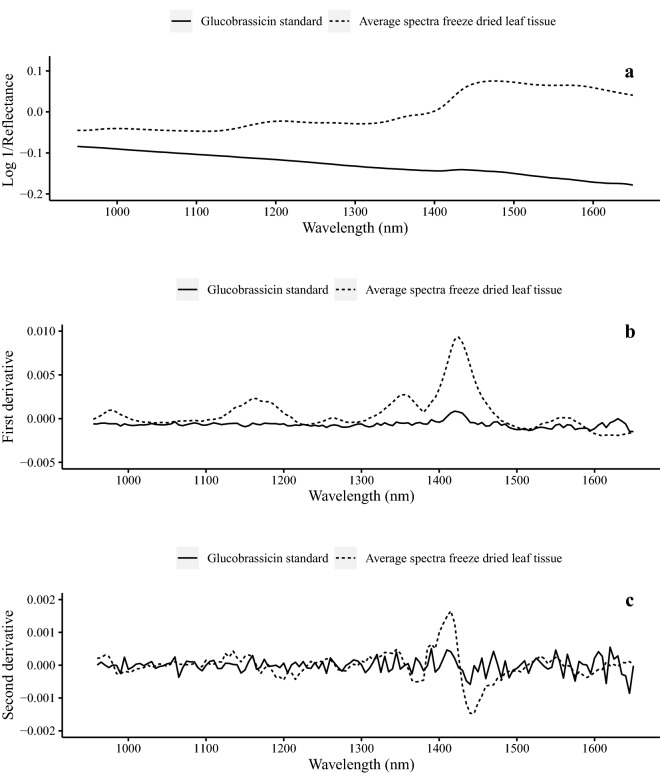
Near-infrared reflectance spectra from a desulphated glucobrassicin (GBS) potassium salt standard (solid line) and the average of all spectra from freeze-dried leaf tissue (n = 92) in the range of 950–1650 nm. Raw reflectance values **a**, first derivative values **b**, second derivative values **c** are represented. Leaf tissue samples were derived from cabbage and Brussels sprouts purchased from grocery stores and natural food cooperatives in Minneapolis and Saint Paul, MN between August and October, 2019

Models were developed using the raw spectral data (log 1/reflectance) as well as on spectra subjected to several preprocessing techniques (Fig. 3). This included (1) taking the standard normal variate (SNV) and de-trending the raw spectral data, (2) taking the first derivative of the raw spectral data, and (3) applying the SNV plus de-trending to the first derivative. The SNV helps compensate for spectral slope and partial size variation while de-trending can remove trends in the spectra [31]. Derivatization can remove the effects of offsets between spectra but does not adjust for baseline slope or scattering effect. Applying the SNV with de-trending to the spectral data has reportedly achieved calibration correlation statistics which were superior to raw or derivative spectra [32]. However, SNV plus de-trending in the present study did not improve calibration statistics, but rather it drastically impaired the model (Table 4). Models we developed on the first derivative of the raw spectral data were very similar to those built with no preprocessing.

**Table 4:** Model statistics for predicting GBS concentration on a fresh weight and dry weight bases

Spectral data used for model development	Calibration (n = 68)		Cross-validation (n = 24)			
	R^2^_cal_^a^	RMSEC^b^	R^2^_CV_^c^	RMSEP^d^	RPD^e^	Terms^f^
Raw (fr wt)	0.89	40.47	0.75	35.12	2.3	10
Raw (dr wt)	0.90	2.17	0.80	2.07	2.4	10
Raw plus SNV + DT (fr wt)	0.65	53.61	0.60	39.56	2.0	2
Raw plus SNV + DT (dr wt)	0.63	3.67	0.41	3.46	1.4	2
1^st^ derivative (fr wt)	0.89	43.29	0.76	30.44	2.6	6
1^st^ derivative (dr wt)	0.90	2.41	0.79	2.07	2.4	6
1^st^ derivative plus SNV + DT^*g*^ (fr wt)	0.76	53.10	0.55	42.17	1.9	2
1^st^ derivative plus SNV + DT (dr wt)	0.64	3.22	0.46	3.33	1.5	1

^*a*^Coefficient of determination of the calibration

^b^Root mean squared error of calibration

^c^Coefficient of determination of the cross-validation

^d^Root mean squared error of prediction

^e^Ratio of prediction to deviation

^f^Number of terms (PLS components) used in the model selected for cross-validation

^g^Standard normal variate with detrending spectral preprocessing

Calibrations equations were developed using a training data set (n = 68) and the equations were internally cross-validated with a testing data set (n_test_ = 24). The data sets were divided using a stratified random sampling procedure so that the testing data set was representative of the training data set. Calibration models were developed by PLSR with a leave-one-out cross-validation method. The leave-one-out method iteratively subsets the training data set into two data sets of with n_train_-1 and 1 observations. To accomplish this, a single data point is removed and acts as a validation data point, and this process occurs iteratively until all observations in the training data set have been used for validation. This processes therefore occurs n_train_ times. The PLS components used in each model were selected using the one-sigma heuristic [33]. This allows the selection of the fewest number of PLS components which are less than one standard error away from the model that fits the data best overall. Component selection is important because incorporating more components results in lower error (RMSEC) but including too many components can result in model overfitting [34]. Spectral data were scaled and centered automatically using the PLS function in R Statistic. Models can be assessed on the cross-validation coefficient of determination (R^2^_CV_) and on the ratio of prediction to deviation (RPD) which is calculated by dividing the standard deviation from the values of the reference method by the root mean squared error of prediction of the cross-validation (RMSEC). Models with RPD values between 2.0 to 2.5 are considered acceptable for qualitative screening into “high”, “medium”, or “low” groups. RPD values greater than 3.0 are considered excellent for quantification, whereas those with values less than 2.0 are generally not useful [35].

### Predicting GBS concentrations

**Fig. 4:**
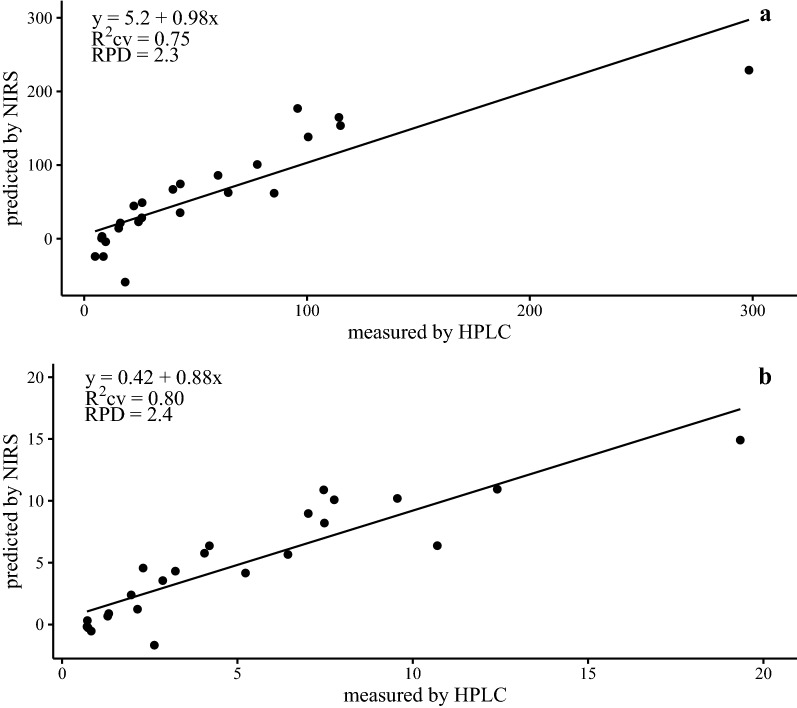
Cross-validation scatter plots (n = 24) of glucobrassicin (GBS) concentration determined using high-performance liquid chromatography and UV-spectroscopy vs. near-infrared reflectance spectroscopy predicted GBS values for **a** μmol GBS·100 g^−1^ fresh weight, and **b** μmol GBS·g^−1^ dry weight. Models were developed from raw spectral data using partial least squares regression

The raw spectral data and the first derivative models outperformed models which applied SNV plus de-trending preprocessing (Table 4). The coefficient of determination of the cross validation (R^2^_CV_) and the residual predictive deviation (RPD) were 0.75 and 2.3 compared to 0.76 and 2.6 for the raw and first derivative spectra respectively when predicting µmol GBS·100 g^−1^ fresh weight. The dry weight basis models were similar to the fresh weight basis models. The R^2^_CV_ and RPD were 0.80 and 2.4 compared to 0.79 and 2.4 for the raw and first derivative spectra, respectively. The number of components included in the model was 10 for those built using the raw spectral data but six for the models built using the first derivative spectra. Since both the raw and derivatized spectra resulted in similar models, the predicted versus measured GBS concentrations using the values derived from the model using the raw spectra are shown in Fig. 4. The RPD values are similar to the SD/SECV metric reported from similar studies and included in Table 2. Both the fresh weight and dry weight basis models could be used to screen samples of cabbage and Brussels sprout leaf tissue for GBS concentration. The model developed here is different from other similar studies in a few important ways. The range of wavelengths used here is smaller than similar studies (950–1650 nm compared to 400–2500 nm) and we used PLSR in R Statistic compared to all other similar studies which used modified PLSR in Win ISI software (Infrasoft International, LLC, Port Matilda, PA). Additionally, the reference chemistry method used here was based on fresh samples and not on freeze-dried powders.

### NIR spectra and loading plots

**Fig. 5:**
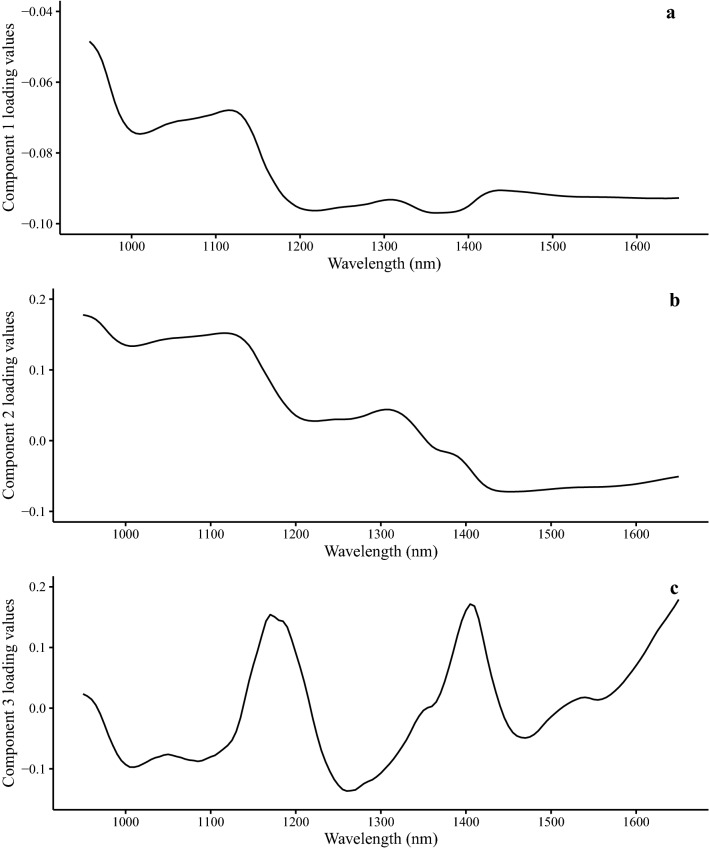
First **a**, second **b**, and third **c** partial least squares (PLS) regression model factor loading values from the raw spectra model for predicting glucobrassicin (GBS) concentration from cabbage and Brussels sprouts. Vegetables were purchased from grocery stores and natural food cooperatives in Minneapolis and Saint Paul, MN between August and October, 2019. PLS regression models were developed to predict both fresh weight and dry weight GBS concentrations. Factor loading values from both models were the same so only one set is represented

Spectral features can be attributed to characteristic molecular vibrations, and these are seen in sample spectra. The features can be interpreted based on chemical knowledge of molecular bands. Calibration loading plots indicate which wavelengths contribute most to each component in the model, and so comparisons between the spectral features and the major wavelengths contributing to the loading plots help define what molecular characteristics are being used for predictions. The average spectra from the freeze-dried leaf tissue compared to the GBS potassium salt standard spectra have similar bands around wavelengths 1420 and 1425 nm (Fig. 3) which corresponds with the first overtone from O–H stretching, but these bands were not clearly present in the loading plots (Fig. 5) of the first three components. The first overtone of the indole N–H asymmetric stretching is associated with a band around 1450 [18]. This was present in all three model component loadings and evident in the average freeze-dried spectra second derivative plot (Fig. 3) and is a distinctive bond in the GBS molecule. However, this region also contains the moisture associated bands of the first overtone from O–H stretching which occurs between 1440–1470 nm [18].

## Conclusions

The models reported here outperform several others [27, 29]. The model developed by Chen et al. [30] performed better than the present study, and it was useful for qualitative determination of GBS in Chinese kale. The model by Chen et al. [30] was built using a wider wavelength range and a greater sample size which could contribute to its better accuracy, although this is not completely clear as there are currently very few studies aimed at the development of GBS calibration models on vegetative tissue. Additionally, the broad, diverse origin of a large number of cultivars that we obtained to develop this model may contribute to higher error compared to that seen by Chen et al. [30]. Arguably, our models would more accurately reflect the diversity of materials breeders, producers, and processors would encounter in utilizing NIRS to characterize GBS concentrations for their particular needs.

NIR spectrometers utilizing the wavelengths between 950 and 1650 nm successfully generated spectra useful in PLSR model development for GBS screening of freeze-dried leaf tissue, a range that is narrower than previously generated NIRS calibration models. Additionally, spectra generated from freeze-dried tissue can be used to successfully estimate GBS concentrations on a fresh-weight basis from a wide variety of cabbages and Brussels sprouts. Predicting GBS concentrations in *Brassica* leaf tissue is possible with the development of calibration models using PLSR applied to NIRS data. Refinement of such models will help accelerate the adoption of GBS-enhancing production practices by allowing producers to verify product quality and thereby achieve premium prices. It will also help increase the scale and efficiency of breeding programs for high GBS-accumulating cultivars. Future work should address which wavelengths or regions of the NIR spectrum are best at predicting GBS concentrations. Doing so could support the development of small, hand-held NIRS devices.

## Materials and methods

### Plant samples

This study was conducted using cabbage (*Brassica oleracea* var. *capitata*) and Brussels sprouts (*Brassica oleracea* var. *gemmifera*) purchased from supermarkets and natural food cooperatives located in Minneapolis and Saint Paul, Minnesota, between August and October, 2019. To capture a wide range of variation in GBS concentrations, samples were selected for variation in size, pigment, and when possible, production location. Our analysis was not concerned with relating specific cultivars or production practices to the GBS concentrations, but rather, we aimed to capture diverse GBS concentrations so that we had a wide sample range from which to develop calibration models that were robust enough to work across many cultivars. To further increase the variability within each sample, cabbage heads were divided into wrapper, inner, and core tissue as we have observed variation among these tissues previously. Plants were used for analysis one to five days after purchase. From previous experiments, the authors have found that GBS concentration in cabbage stored at 98% relative humidity and 2º C was not significantly reduced over three months (Fritz, unpublished data). Each sample was split into two parts, one part to be used for wet chemistry analysis and the other for NIRS. The sample was chopped to approximately 2 cm square pieces and mixed to ensure homogeneity. Half of the sample was flash frozen for later lyophilization, and the remaining half was immediately placed in boiling water to begin sample preparation for HPLC analysis.

Chemical quantification of GBS. GBS was quantified as described by Hecht et al. [36] using modifications from Rosen et al. [37]. Samples of 100 to 150 g fresh weight were boiled in water in a volume of water three-times their weight for five minutes to deactivate myrosinase. Samples were cooled at room temperature for ten minutes, then macerated in a blender for two minutes, then a 40-mL aliquot of blended sample was stored at − 30˚ C until further analysis which occurred within 30 days. Later, samples were thawed and homogenized for two minutes at 12,000 rpm with a Polytron PT 1300 D homogenizer (Kinematica AG, Lucerne, Switzerland), and then 2-mL sample of the homogenate was centrifuged for 4 min at 8000 g at 4˚ C.

Desulfonated GSLs were extracted from the supernatant with solid phase strong anion exchange (SAX) columns (Sigma-Aldrich, St. Louis, MO). In a vacuum manifold, SAX columns were washed with 2 mL of 0.50 M sodium acetate buffer (pH 4.6), followed by 2 mL of deionized water. 500 µL of supernatant from centrifuged samples were filtered through columns followed by 1 mL of 0.02 M sodium acetate (pH 4.0) buffer. Finally, 1 mL of 0.2 mg·mL^−1^ sulfatase solution (aryl-sulfate sulfohydrolase from Helix pomatia—Type H-1; Sigma-Aldrich, St. Louis, MO) was vacuum-infiltrated through the columns. Columns were incubated for ~ 15 h at room temperature before elution with 3 mL of water (elution occurred in two steps, first with 2 mL and then with 1 mL) and collected volumes were determined by weight. Eluents were stored at − 30˚ C until HPLC analysis which occurred within 14 days.

HPLC analysis was carried out using an Agilent 1200 Series Quaternary system (Agilent Technologies, Inc., Santa Clara, CA), with the diode array detector set at λ = 229 nm, using a Luna C18, 5 µm, 250 × 4.6 mm guard column (Phenomenex, Torrence, CA) set at 30 °C. 50 μL of eluent was injected and separated on the column with the following flow rates and gradients: 0–2 min gradient 5–15% acetonitrile, 1 mL/min; 2–20 min gradient 15–47% acetonitrile, 1 mL/min; 20–22 min gradient 47–100% acetonitrile, 1–1.15 mL/min; 22–26 min, 100% acetonitrile, gradient 1.15 to 1.3 mL/min; 26–28 min, 100% acetonitrile, gradient 1.3–1.5 mL/min; 28–35 min, 5% acetonitrile, 1 mL/min. GSL peaks were viewed in OpenLAB Chromatography Data System with rev. C.01.06 software and GBS was identified based on retention time. Concentration was determined using sinigrin as an external standard and previously published response factors [38].

Near-infrared reflectance spectroscopy (NIRS) analysis. Plant samples of 80 to 150 g were wrapped in aluminum foil, flash frozen in liquid nitrogen, placed on dry ice, and stored at −80° C (S-7805, Uline, Pleasant Prairie, WI) until lyophilization. Frozen samples were placed inside a 35 L VirTis 24Dx48 general purpose freeze dryer (SP Scientific, Stone Ridge, NY, USA) which was kept at −30° C for seven days, then after one week the temperature was raised to −20 °C, 24 h later it was raised again to −10 °C, and 24 h later the temperature was then allowed to slowly raise over 3 days until it achieved room temperature and samples were then removed. Freeze-dried samples of ~ 10 g were pre-ground in a 12-cup electric coffee grinder and further ground using a Retsch ZM200 (Retsch group, Haan, Germany) grinder with 35 mesh (0.5-mm particle size). Between grinding different samples, the mesh was removed and cleaned with a brush and pressurized air to avoid cross contamination. Samples were stored in small coin envelopes (S-14719, Uline, Pleasant Prairie, WI), packed with indicating silica gel desiccant packets (MiniPax absorbent packets, Sigma-Aldrich, Saint Louis, MO), and stored inside an airtight container with for up to two weeks prior to NIRS analysis.

Diffuse reflectance NIRS was applied using a diode array NIRS instrument (DA 7250 NIR Analyzer; Perten Instruments, Hägersten, Sweden). For each NIRS scan, a 10-g sample of freeze-dried cabbage or Brussels sprout leaf tissue was placed in a 22-ml volume NIRS sample dish by pouring the powdered sample into the dish and using a straight edge to scrap off any extra sample so that the surface was consistent across all scans. The minimum quantity of freeze-dried sample needed for each NIR scan was related to the size of the sample dish. For our samples, approximately 10-g of leaf tissue was sufficient to slightly overfill the sample dish so excess could be scraped off. Each of the 92 samples were scanned twice for a total of 184 scans. The average spectrum of each induvial sample was calculated. The calibration models were developed using the average spectrum of each of the 92 individual samples. NIRS scans were taken in the wavelength range of 950 to 1650 nm at an interval of 5 nm. Testing and training data sets were split using a stratified random sampling procedure to split the data into calibration (n = 68) and validation (n = 24) data sets. Calibration and validation were performed with R Statistical software version 3.2.4 [39] using the pls package [40]. An additional data file generated for chemometrics are included [Additional file 1].

## Supplementary information

**Additional file 1.** Description of data: NIRS spectra between 950 and 1650 nm recorded as the log of 1/reflectance.

## Data Availability

All data generated or analyzed during this study are included in this published article [and its supplementary information files [see additional file: NIR_GBS_Chemometrics.xls].

## Abbreviations

GBS: Glucobrassicin
GSL: Glucosinolate
HPLC: High-performance liquid chromatography
NIRS: Near infrared spectroscopy
PLSR: Partial least squares regression
R^2^_cal_: Coefficient of determination of calibration
R^2^_cv_: Coefficient of determination of cross-validation
RMSEP: Root mean squared error of prediction
RMSEC: Room mean squared error of calibration
RPD: Ratio of prediction to deviation
SECV: Standard error of cross-validation
SNV: Standard normal variate
